# Multiple Merkel cell carcinoma: a case report

**DOI:** 10.1093/omcr/omad081

**Published:** 2023-08-20

**Authors:** Abdullah Omar, Houda Alassil, Duha Alloush

**Affiliations:** Department of Dermatology and Venerology, Damascus Hospital, Damascus, Syria; Faculty of Medicine Syrian Private University, Damascus, Syria; Department of Dermatology and Venerology, Damascus Hospital, Damascus, Syria; Department of Dermatology and Venerology, Damascus Hospital, Damascus, Syria

## Abstract

Merkel cell carcinoma (MCC) is an uncommon, fast-growing tumour, classified as a neuroendocrine neoplasm that affects the skin and spreads quickly to other parts of the body. It often affects aged, immunosuppressed people with a history of sun exposure and has a slight tendency to affect men. This report represents a case of multiple MCC nodules distributed on the chest and head of a 65-year-old Syrian male. The lesions occurred 1 year before presentation. The excisional biopsy confirmed the diagnosis of MCC. Further investigations indicated to assess metastasis, but the patient passed away before completing our recommendation. In this report, we aim to confirm the vital role of early detection when dealing with an aggressive tumour such as MCC, with a 50% chance of survival for 5 years. Otherwise, more lesions will worsen the prognosis, precisely like what occurred with our patient.

## INTRODUCTION

Merkel cell carcinoma (MCC) or primary neuroendocrine carcinoma of the skin is an uncommon, fast-growing tumour, classified as a neuroendocrine neoplasm that affects the skin and metastasises to other parts of the body, especially in older, immunosuppressed people. The origin of this disease is controversial; probable cells of origin are the Merkel cells (touch cells) based on similarities in morphologic, immunohistochemical and ultrastructural features and early B-cells based on cellular morphology. It usually presents as a rapidly growing, pink–red to violaceous coloured, firm, solitary nodule; it affects sun-exposed areas such as the head and neck [1].

Diagnosis is made based on clinical features and biopsy with immunohistochemistry. MCC should be considered in any tumour with ‘AEIOU’ clinical features (A: asymptomatic, E: expanding rapidly, I: immune suppressed, O: older than 50, U: UV (Ultraviolet)-exposed fair skin). Immunohistochemistry plays a significant role in the diagnosis of CK20 (Cytokeratin) staining. Early aggressive dealing is necessary, often with surgery, radiation therapy and chemotherapy [2].

## CASE REPORT

A 65-year-old man presented to the dermatology clinic with rapidly developing, multiple, painful pink nodules; the lesions accounted for ~20 lesions on the trunk (chest, back) and the temporal region. The masses appeared 1 year before the presentation.

**Figure 1:**
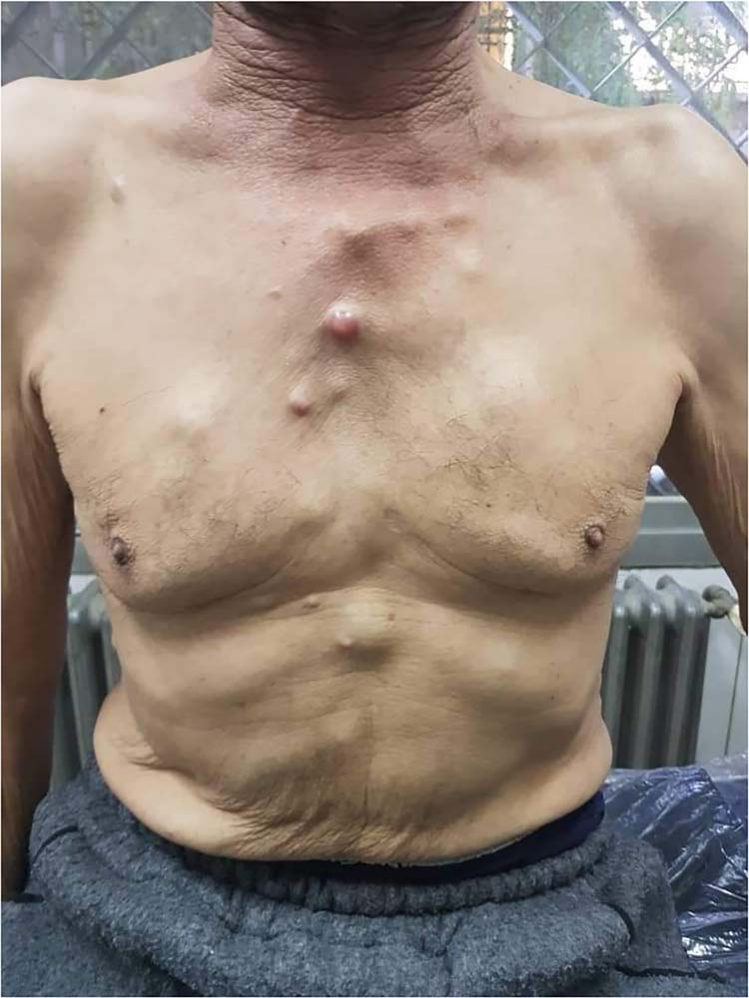
A total of 11, irregular nodules on the chest and epigastric region; note the neck solar elastosis triggered by chronic sun exposure.

By examining the nodules, they were tender, not movable at the skin and fixed deeply to the inner tissue layer. The masses’ measurements varied from 5 to 20 mm in diameter (Fig. 1).

The patient suffered from a chronic cough and fatigue in addition to no measured weight loss and loss of appetite.

**Figure 2:**
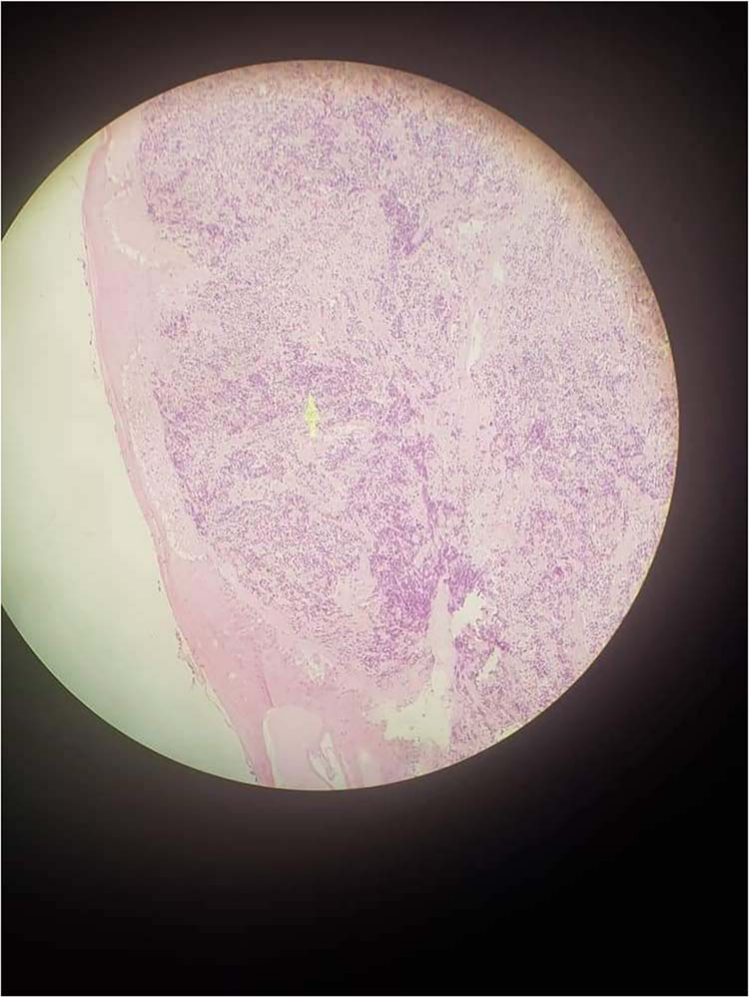
Uniform, small and round cells with scant cytoplasm with domination of nests and trabecular appearance.

The patient was prepared for an excisional biopsy; the histopathological examination showed a lobulated appearance of small, round and blue cells by microscopy (Fig. 2). The differential diagnosis involved MCC and metastasis from pulmonary small cell carcinoma. The biopsy underwent immunohistochemistry to confirm the diagnosis. The stain showed positivity for cytokeratin and chromogranin in addition to the CK20 positivity. The patient was sent to the oncology department to continue assessing the medical case. The patient underwent treatment with carboplatin and etoposide while in a late stage. Unfortunately, the prognosis was bad, and the patient died after a couple of weeks, although he was undergoing chemotherapy.

## DISCUSSION

MCC usually presents as a pink–red to violaceous, firm, dome-shaped, solitary nodule that proliferates rapidly [3]. Suk *et al*. [3] described a single, painless, firm, erythematous and rapidly growing nodule on the left cheek area of a 77-year-old woman. Coggshall *et al*. [4] confirmed that MCC is symptomatic and tender in most cases, while Becker *et al*. [5] declared that MCC most commonly presents as an erythematous or violaceous, tender, indurated nodule accounting 209 occurring on sun-exposed areas on the head or neck of an elderly Caucasian man. Confirmation of diagnosis relies on analyses of histological features and immunological marker expression profiles of the lesion [4]. In our case, the tumour was tender and multiple about 20 lesions, and we confirmed MCC diagnosis by the immunohistochemistry tests, which were positive for cytokeratin, chromogranin and CK20. CK-20 is low-molecular-weight ker­atin typically expressed in the gastrointestinal epithelium and some gastrointestinal and transitional cell carcino­mas. CK-20 was the first immunostaining technique used to diagnose MCC reliably [3]. MCC tumours are usually CK-20-positive, although it is essential to note that CK-20 expression has been reported to be negative in 5–10% of MCC cases [3]. *Chromogranin* is a secretory protein found in the large dense-core vesicles of neuroendocrine cells. The Chromogranin A assay appears reliable for diagnosing and following up on various endocrine tumours (principally pheochromocytomas, neuroblastomas, gastrinomas and carcinoid tumours) [6]. According to the late attendance of the patient, he was reviewed with a late tumour stage, which is why surgery was not a therapeutic choice as the patient had a noticeable weight loss and cachectic; this condition suggests a metastatic tumour. We recommend a computed tomography scan for confirmation. The rapidly progressive state impeded the verification when our patient passed away. Most of the published cases did not discuss metastasis chemotherapy management because of the rarity of the disease [7]. Chemotherapy is reserved for metastatic disease and is similar to the treatment of lung small-cell carcinoma because of the exact neuroendocrine origin with etoposide and topotecan [7]. Our patient underwent treatment with carboplatin and etoposide under the oncologist’s supervision. Several publications have appeared in recent years documenting the increased incidence of MCC over the past 30 years. Coggshall *et al*. [4] recommend increased reporting and improvements in diagnostic techniques to explain this raise, so our case resembles the participation of a developing country lacking in reported cases to reveal this vague.

## CONCLUSION

This case report is one of a kind in Syria; we focused on the possibility of encountering such a fatal malignancy and how to do our best—with our limited resources—to deal with it.
